# Supplementary material for: Increasing Silver Nanowire Network Stability through Small Molecule Passivation
Source: Nanomaterials (Basel). 2019 Jun 20;9(6):899. doi: 10.3390/nano9060899 (PMC6631436; doi:10.3390/nano9060899)
Supplement: Supplementary file 1 [file nanomaterials-09-00899-s001.pdf]

Supplementary Materials: Increasing Silver Nanowire Network Stability through Small Molecule Passivation

Alexandra Madeira [1], Marie Plissonneau [1], Laurent Servant [2], Irene A. Goldthorpe [3] and Mona Tréguer-Delapierre [1,*]

Table of Content

1. Comparison of the performances of AgNW films passivated before and after their formation into films (Figure S1 and Table S1)
2. Chemical analysis of a MUA-coated AgNW network (32 nm) (Figures S2 - S4)
3. Chemical stability of a MUA-coated AgNW network after storage in the dark (Figure S5)
4. Characterization of PVP or MUA-coated AgNW networks exposed to daylight (Figures S6 and S7)
5. Chemical stability of the network of Ag nanowires of 70 nm (Figures S8 and S9)
6. Chemical stability of the AgNW networks coated with PPh_3 or MUA (Figure S10)

1. Comparison of the performances of AgNW films passivated before and after their formation into films

Table S1. Initial sheet resistance (Rs) and transparencies (T) of the electrodes based on silver nanowires passivated before or after their formation into films.

Parameters	MUA-Passivated In solution	MUA-Passivated After their formation into electrodes
Rs (Ω/Sq)	15.5 ± 2.0	17.0 ± 2.0
T	56 ± 3%	71 ± 2%

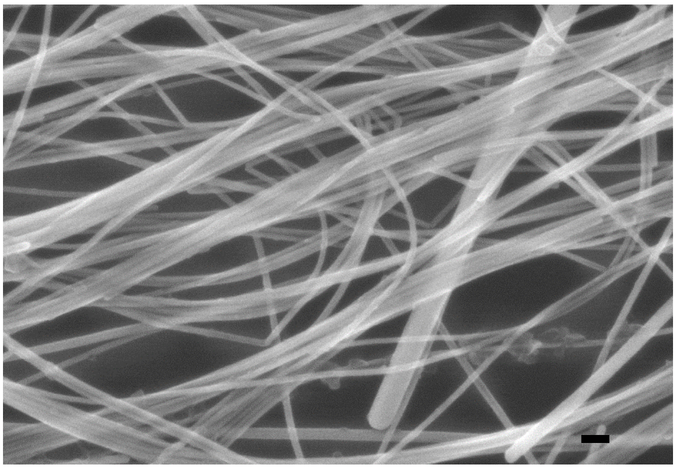

Figure S1. SEM image of AgNW passivated with MUA in solution and subsequently deposited as a film (scale bar: 100 nm). The MUA-coated AgNWs have a lower colloidal stability than the PVP-coated ones. Large agglomerates are formed in solution and affect the transparency of the film.

2. Chemical analysis of a MUA-coated AgNW network (32 nm)

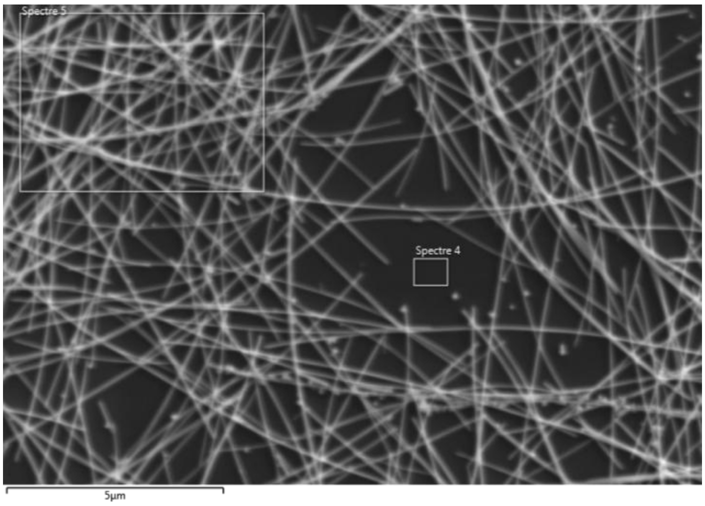

(**a**)

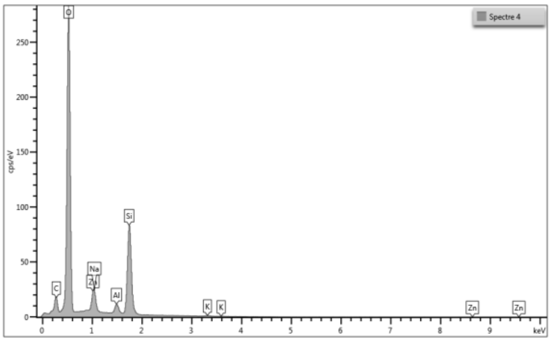

(**b**)

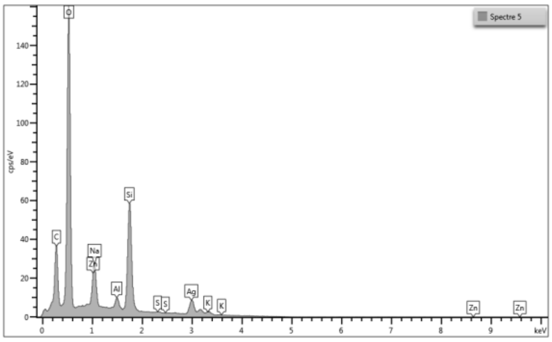

(**c**)

Figure S2. (**a**) SEM image of a MUA-coated silver NW network. Representative EDX spectrum taken on different areas of the networks: (**b**) area without AgNWs; (**c**) area with AgNWs. No traces of MUA were found on the areas without nanowires.

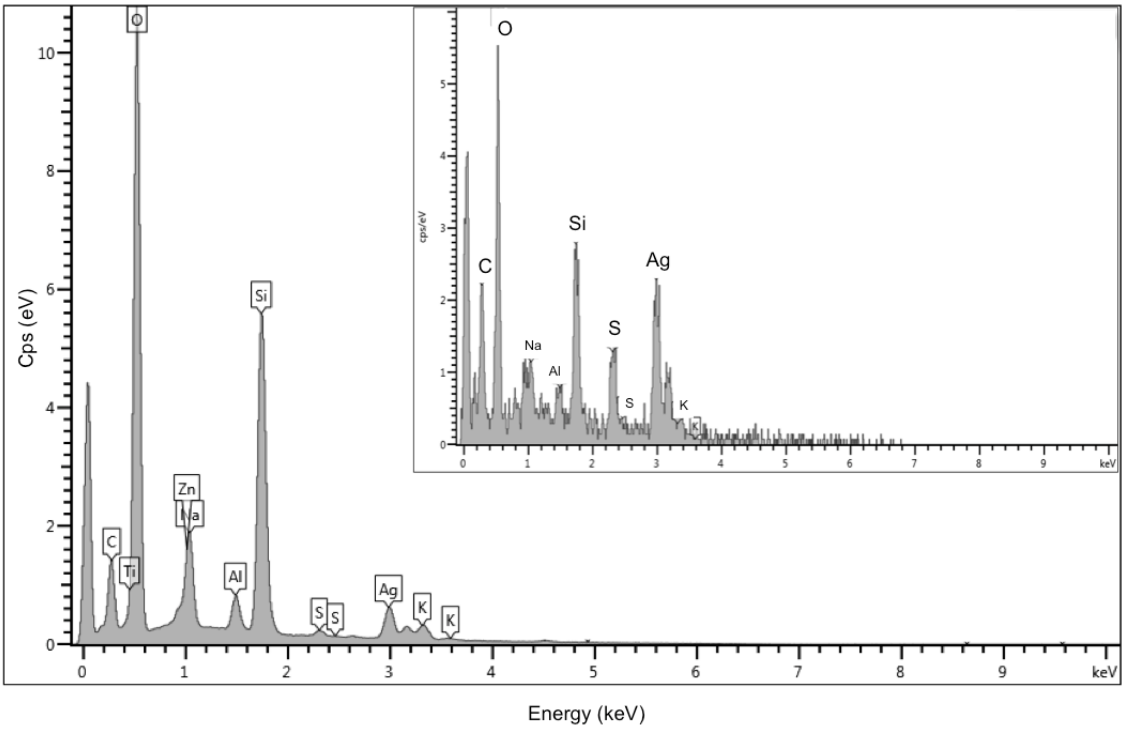

Figure S3. Typical EDX spectrum taken from an area with Ag nanowire of a MUA-coated silver NW network. Inset: Magnification of the spectrum around the S peaks.

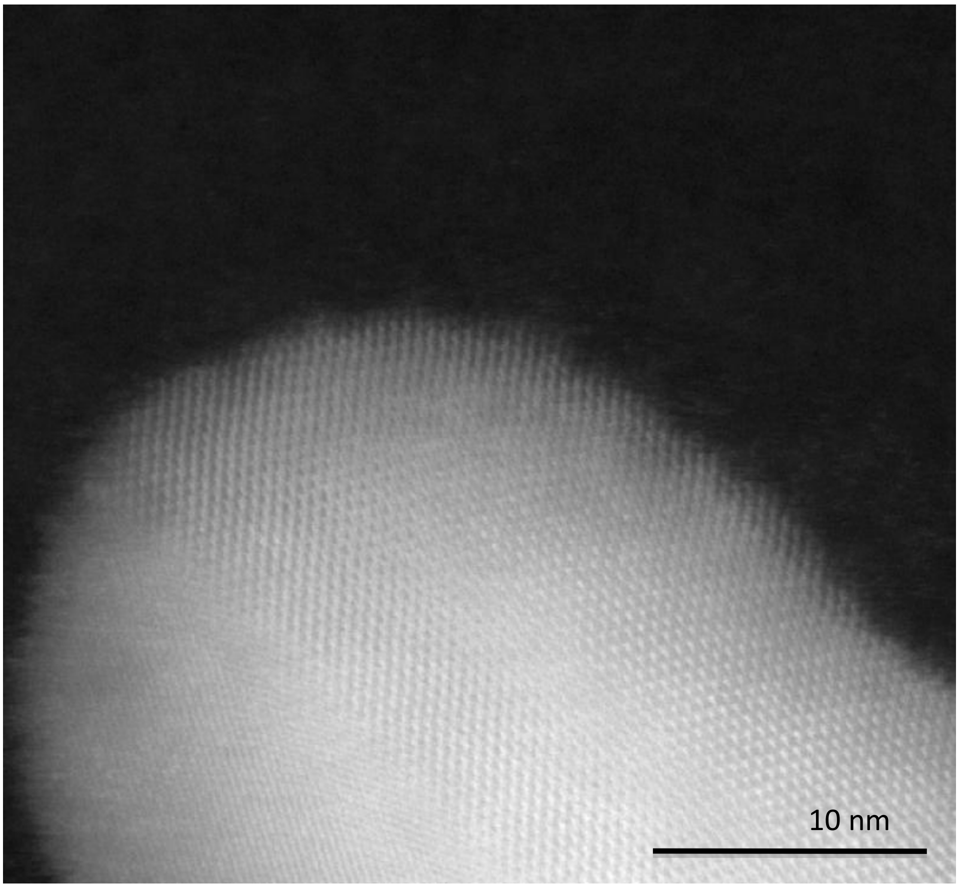

Figure S4. Atomic Resolution Microscopy image of a MUA-coated silver nanowire.

3. Chemical stability of a MUA-coated AgNW network after storage in the dark

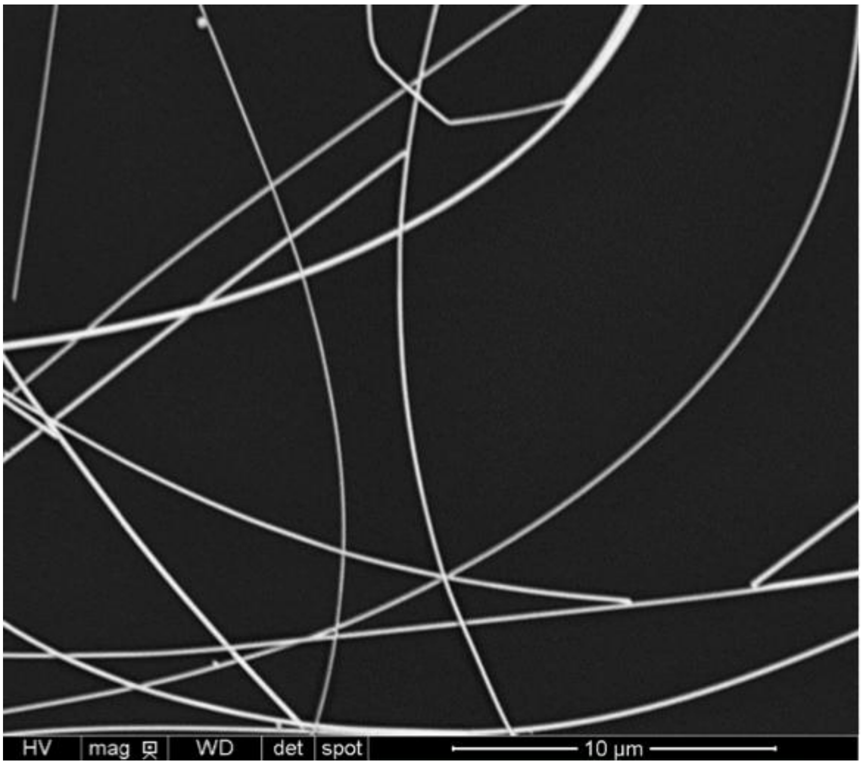

Figure S5. SEM image of a MUA-coated silver NW network stored and exposed to the dark after 4 months.

4. Characterization of PVP or MUA-coated AgNW networks exposed to daylight

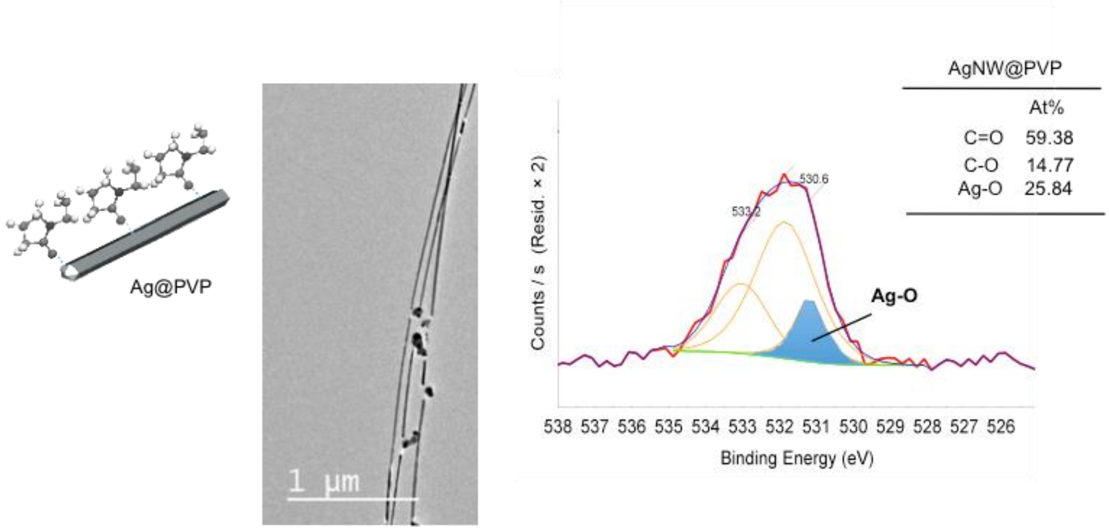

Figure S6. TEM image and XPS spectrum of the PVP-coated AgNWs after 4 months of air exposure (O 1s region scan).

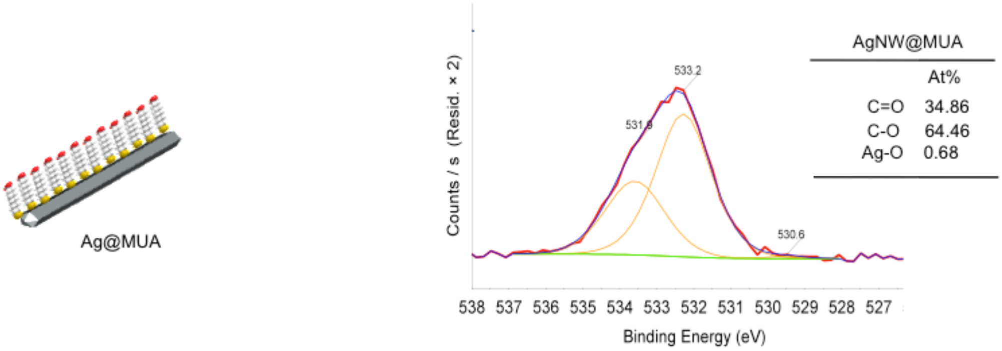

Figure S7. XPS spectrum of the MUA-coated AgNW network stored and exposed to daylight after four months (O 1s region scan).

5. Chemical stability of the network of Ag nanowires of 70 nm diameter

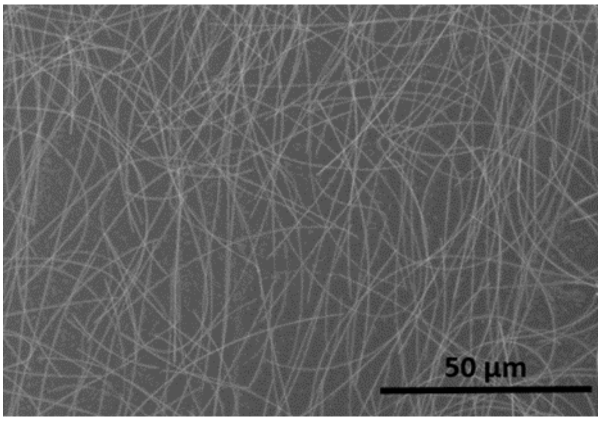

Figure S8. SEM image of 70 nm diameter Ag NW network.

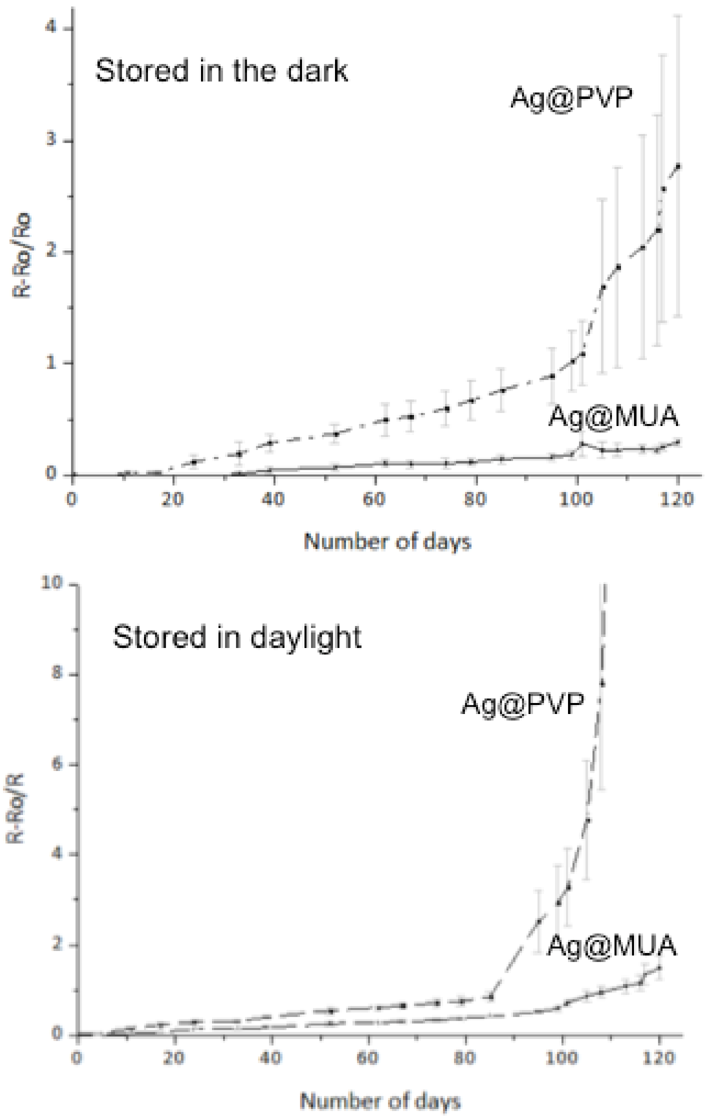

Figure S9. Evolution of the sheet resistance vs time for films of PVP- or MUA-coated AgNW (70 nm) after exposure to air at ambient conditions.

6. Chemical stability of the network of Ag nanowires coated with PPh_3 or MUA

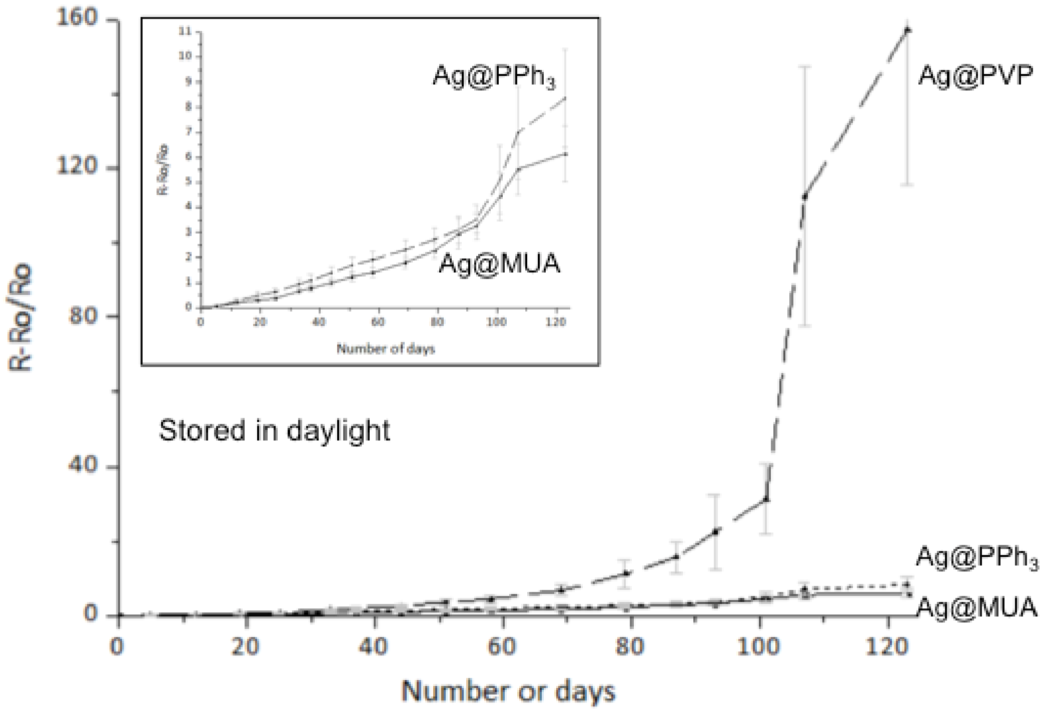

Figure S10. Evolution of the sheet resistance vs time for films of AgNW (32 nm diameter) coated with PVP, MUA and PPh_3 stored in day light after exposure to air at ambient conditions**.** Inset: magnification of the graphs for MUA- or PPh_3– coated AgNW network.
